# Editorial: Intermittent feeding in critically ill patients

**DOI:** 10.3389/fnut.2023.1295405

**Published:** 2023-10-16

**Authors:** Cristian Deana, Pietro Vecchiarelli, Edoardo Picetti, Alessio Molfino

**Affiliations:** ^1^Department of Anesthesia and Intensive Care, Health Integrated Agency of Friuli Centrale, Udine, Italy; ^2^Intensive Care Unit, Ospedale Belcolle, Viterbo, Italy; ^3^Department of Anesthesia and Intensive Care, Parma University Hospital, Parma, Italy; ^4^Department of Translational and Precision Medicine, Sapienza University of Rome, Rome, Italy

**Keywords:** enteral nutrition, critically ill patient, parenteral nutrition, intermittent feeding, autophagia, refeeding syndrome, proteins

Critical illness deeply alters the patients' metabolism, determining a high catabolic state since the early phase of the intensive care unit (ICU) stay. Thus, the risk to develop toward malnutrition is high especially if an adequate artificial nutrition therapy is not provided (1).

However, we are far from having available a unique nutritional prescription able to “fit” all in an ICU setting. We should consider that artificial nutrition may confer benefit to the patients, but also could be harmful, if not adequately and timely delivered.

One of the main aspects that makes artificial nutrition complex in ICU is represented by the heterogeneous population admitted for the different acute diseases. Sir Cuthbertson in the 50's described different phases of critically illness, with a non-inhibitable catabolic state at the initial phase of the disease, then becoming anabolic only when the storm has passed. Unfortunately, we have no precise markers for the transition from one phase to another.

For this reason, clinical trials that started early full nutrition in order to reduce the catabolism failed to provide clinical benefits. Indeed, researchers observed that an excess of calories and proteins too early in less severe ICU patients may increase mortality rates (2).

In this regard, the amount of proteins plays a key role during the early phase of critically ill patients: some large randomized trials demonstrated no advantage on mortality with higher compared with lower protein nutrition content (3). Furthermore, evidences suggest that high protein load administered too early could impair autophagy, a protective mechanism that removes damaged cells (4).

The route by which artificial nutrition is delivered is another important point. The use of early parenteral nutrition (PN) was revisited after the EPANIC trial in whom patients that received late (after 7 days from ICU admission) PN had faster recovery, fewer complications and indirectly produced lower health related costs (5).

However, when PN has to be started, for example when enteral route is contraindicated, some advices have to be considered to reduce potential complications, such as catheter-related blood stream infections and thrombosis (Zaccone et al.; Ko et al.).

Enteral nutrition is nowadays the preferred route of feeding critically ill patients, contributing to maintain a trophic intestinal mucosa. Continuous enteral feeding is widely used because it is easier and require less effort for personnel.

However, it does not reproduce the physiologic response to muscle protein synthesis after protein bolus. Moreover, continuous tube feeding abolishes the entero-hormonal response. Intermittent feeding could be better compared to continuous feeding considering that it can enhance muscular protein synthesis, and the fasting periods may be protective through enhanced autophagy and ketogenesis (6).

Moreover, both methods do not prevent gastrointestinal complications, such as diarrhea or constipation [(7), Qu et al.].

An important aspect to consider when initiating artificial nutrition after a prolonged fasting state is the refeeding syndrome. Low phosphate levels are the main characteristic of this complication, but other electrolytes disturbances should be taken into consideration.

In neurocritically ill patients, often necessitating enteral nutrition due to swallowing disturbances, some risk factors, among them APACHE II score, SOFA score, low serum albumin and low circulating levels of potassium, have been indicated by Zhang et al..

Sepsis is another severe condition where deep disturbances in metabolism are often observed. Evidences pointed out that vitamin C plus hydrocortisone and thiamine reduced mortality in septic patients (8). However, Liang et al. in their meta-analysis did not confirm this aspect, suggesting new studies investigating this important topic.

Guan et al. in a retrospective study including more than 19,000 patients, found that vitamin D supplementation in ICU patients reduced the risk for sepsis and new mechanical ventilation, but not mortality at 28-day.

Based on several evidences, an ideal nutrition is far from actual practices. However, moving toward precise and personalized medicine also among ICU patients, probably using in the next years artificial intelligence models, represents an intriguing scenario.

Also, the continuum of care, from the hospital to the rehabilitation units and finally in a home setting, should be effective in reducing the incidence of malnutrition with its negative long-terms effects after a critical illness [(9); Diamanti et al.].

## Author contributions

CD: Writing—original draft, Writing—review and editing. PV: Writing—original draft, Writing—review and editing. EP: Writing—original draft, Writing—review and editing. AM: Writing—original draft, Writing—review and editing.
